# Cognitions in bipolar affective disorder and unipolar depression: imagining suicide

**DOI:** 10.1111/j.1399-5618.2011.00954.x

**Published:** 2011-11

**Authors:** Susie A Hales, Catherine Deeprose, Guy M Goodwin, Emily A Holmes

**Affiliations:** Department of Psychiatry, University of OxfordOxford, UK

**Keywords:** bipolar disorder, cognitive therapy, depression, imagery, risk assessment, suicide

## Abstract

**Objective:**

Bipolar disorder has the highest rate of suicide of all the psychiatric disorders. In unipolar depression, individuals report vivid, affect-laden images of suicide or the aftermath of death (flashforwards to suicide) during suicidal ideation but this phenomenon has not been explored in bipolar disorder. Therefore the authors investigated and compared imagery and verbal thoughts related to past suicidality in individuals with bipolar disorder (n = 20) and unipolar depression (n = 20).

**Methods:**

The study used a quasi-experimental comparative design. The Structured Clinical Interview for DSM-IV was used to confirm diagnoses. Quantitative and qualitative data were gathered through questionnaire measures (e.g., mood and trait imagery use). Individual interviews assessed suicidal cognitions in the form of (i) mental images and (ii) verbal thoughts.

**Results:**

All participants reported imagining flashforwards to suicide. Both groups reported greater preoccupation with these suicide-related images than with verbal thoughts about suicide. However, compared to the unipolar group, the bipolar group were significantly more preoccupied with flashforward imagery, rated this imagery as more compelling, and were more than twice as likely to report that the images made them want to take action to complete suicide. In addition, the bipolar group reported a greater trait propensity to use mental imagery in general.

**Conclusions:**

Suicidal ideation needs to be better characterized, and mental imagery of suicide has been a neglected but potentially critical feature of suicidal ideation, particularly in bipolar disorder. Our findings suggest that flashforward imagery warrants further investigation for formal universal clinical assessment procedures.

Suicide is a major cause of death in the modern world and, of all the psychiatric disorders, bipolar disorder confers the highest risk of completed suicide (1). The assessment of suicide risk is arguably one of the primary skills we expect clinicians to acquire and when doing so, we ask questions such as “Do you have thoughts about killing yourself?; do you have a plan?”. Asking about *thoughts* typically evokes a response about suicidal ideation in a narrative form. The experience of corresponding mental imagery is not usually elicited at all. However, we know that cognitions in the form of mental images have a more powerful emotional impact than their verbal counterparts (2). Suicidal patients may employ not only a narrative to reflect on suicide but also simulate in images how to complete it (3–5).

Individuals with unipolar depression have been found to experience repetitive suicide-related images at the time they are most despairing or suicidal (3, 5). These mental images of future suicidal acts have been termed flashforwards, to emphasize the analogy with imagery *flashbacks* to past trauma. Flashforwards may be particularly important because the act of *imagining* future events has been found to be causal in predicting behaviour (6), and therefore could increase the likelihood of completing a suicidal act. To give a single example from our clinic, one male patient with bipolar I disorder reported persistent, intrusive vivid imagery of himself jumping from a certain cliff when suicidal. He had a history of escaping the inpatient ward to try to reach this cliff, which was some distance from the hospital (3). There are also literary accounts of bleak, death-laden imagery in bipolar depression (7) and vivid mental imagery appears to be present in acute depressive and manic phases of bipolar disorder (cf., 8, 9–12). However, the occurrence of suicidal imagery is yet to be formally explored in this population, despite the elevated risk of suicide. As we have proposed that mental imagery acts as an emotional amplifier in bipolar disorder (13), the aim of our study was to provide a first test of whether suicidal imagery is present in bipolar disorder. In addition we sought to replicate previous findings of suicidal flashforwards in unipolar depression. The second aim of the study was to compare the qualities of any such imagery between bipolar and unipolar depressed groups. Specifically, we hypothesized that there may be increased preoccupation with flashforward imagery in participants with bipolar disorder, as predicted by their greater trait propensity to be visualizers than verbalizers (13). Further, given that impulsivity is a characteristic of bipolar disorder, we hypothesized that the perceived likelihood of acting on any suicidal imagery (i.e., the *compellingness* of the imagery) would be elevated in the bipolar sample (14).

## Participants and methods

This was a quasi-experimental comparative study: bipolar disorder versus unipolar depression.

### Participants

#### Bipolar disorder group

Twenty people with bipolar disorder and a history of past suicidal ideation (12 with bipolar I disorder, 7 with bipolar II disorder, and 1 with cyclothymia) were recruited from clinic attenders in secondary care and the community. Potential outpatients were approached by their consultant psychiatrist or community mental health team keyworker. Adverts were placed in local education and community centres, on local and national internet sites, and in the service user magazine *Pendulum*.

Advertisements stated that the researchers were “investigating mental processes at times of utmost crisis in people with experience of low mood, depression or bipolar disorder”. There was no specific reference to mental imagery.

The inclusion criteria were: a DSM-IV (15) diagnosis of bipolar disorder (bipolar I disorder, bipolar II disorder, cyclothymia, or bipolar disorder not otherwise specified); a score greater than 0 on items 4 and 5 of the Beck Suicide Scale–worst ever version (BSSw) (16), indicating past active suicidal ideation; age 18–65; ability to read and write English; and willingness to give written informed consent to participate in the study. Diagnoses were confirmed by Structured Clinical Interview for DSM-IV (SCID) (17). Of the bipolar group, 14 met criteria for a current major depressive episode. Current depressed mood was assessed using the Beck Depression Inventory (BDI-II).

#### Unipolar disorder group

Twenty people with unipolar depression and past suicidal ideation (10 with major depressive disorder, 3 with major depressive disorder in partial remission, and 7 with dysthymic disorder) were recruited using similar strategies. The inclusion criteria were: a recent [within the past 12 months (18)] or current DSM-IV major depressive episode (MDE) or dysthymic disorder according to the SCID (17); a score above the cut-off for mild depression (>14) on the BDI-II (19), indicating current depressed mood; a history of suicidality on the BSSw (16) (as above); and no lifetime history of mania or hypomania. Other inclusion criteria remained the same as those above. Of the unipolar group, 10 met criteria for a current major depressive episode.

Individuals were not entered into the study if they had a current episode of mania or hypomania (bipolar group) or current severe suicidal ideation [defined as (i) a score of ≥ 1 on the Beck Suicide Scale–current version (16) screening questions and (ii) judged to be at risk of an imminent suicide attempt as determined by the University of Oxford risk assessment protocol].

### Procedures

The study was approved by a National Health Service Research Ethics Committee. After description of the study, written informed consent was obtained from all participants. Once diagnoses had been confirmed with the SCID, participants completed the BDI-II (19), Altman Mania Rating Scale (AMRS) (20), Spielberger State-Trait Anxiety Inventory–State version (STAI-S) (21), and BSSw (16). Those who met inclusion criteria were invited to take part in the experimental phase of the study. They completed the Suicidal Cognitions and Flashforwards interview (3), Spontaneous Use of Imagery Scale (SUIS) (22), Impact of Future Events Scale (IFES) (23), Barratt Impulsiveness Scale (BIS) (24), and National Adult Reading Test (NART) (25) to check intellectual comparability between groups. Participants were debriefed and received £10 reimbursement.

### Psychological measures

#### Beck Suicide Scale–worst ever version (BSSw) (16)

The BSSw consisted of the first 19 items of the Beck Scale for Suicide Ideation (26), worded in the past tense. Respondents answered the items based on the time that they felt most depressed about life. The first five items were screening questions. If a participant scored greater than zero on question four or five of the scale this indicated significant suicidal ideation, and they were required to complete the measure.

#### Suicidal Cognitions and Flashforwards Interview (3)

The Suicidal Cognitions and Flashforwards Interview is a structured clinician-administered instrument which assesses the content and qualities of verbal thoughts and mental images (flashforwards) (3) related to suicide. Questions were anchored to the time when participants felt at their most despairing or suicidal. The difference between verbal thoughts (e.g., sentences) versus sensory mental images (e.g., pictures) was clarified. Participants were asked whether they experienced any suicide-related imagery and also any suicide-related verbal thoughts at their worst times. A checklist of typical categories was used to structure the interview (e.g., ‘Planning/preparing to harm yourself or make a future suicide attempt’, ‘Things you were escaping from’, ‘What might happen to others if you died’). Next, participants made four separate ratings on 9-point Likert scales for (i) time spent preoccupied with suicide-related imagery, (ii) time spent preoccupied with suicide-related verbal thoughts, (iii) compellingness of suicide-related imagery, and (iv) compellingness of suicide-related verbal thoughts (for preoccupation: 1 = none of the time to 9 = all the time; for compellingness: 1 = not at all compelling to 9 = completely compelling). Finally, participants selected their most significant suicidal flashforward image and described it in detail. They were asked: *“What did the image make you want to do?” (response) and “What did the image mean to you?” (appraisal).* Ratings of (i) distress associated with the flashforward and (ii) comfort associated with the flashforward were made on separate 9-point Likert scales (1 = not at all distressing/comforting to 9 = extremely distressing/comforting). The interview was audio-taped and transcribed verbatim.

#### Spontaneous Use of Imagery Scale (SUIS) (22)

The SUIS provided a trait measure of use of (non-emotional) imagery in everyday life. A series of descriptions was given, for example: “When I think about visiting a relative, I almost always have a clear mental picture of him or her”. Each description was rated on a 5-point scale, from 1 = never appropriate to 5 = always completely appropriate.

#### Impact of Future Events Scale (IFES) (23, 27)

The IFES provided an index of the impact of intrusive prospective imagery. Participants were first asked to “*Please identify three future events which you have been thinking about by imagining over the past seven days (e.g., positive or stressful life events). For each event, please indicate whether your imagining of it was positive or negative*”. This first step was to encourage participants to respond on the IFES in relation to their specific and idiosyncratic future events. Participants then completed 24 items to assess intrusive pre-experiencing, avoidance, and hyper-arousal with the instructions “*Below is a list of comments made by people about imagining events in the future. Please read each item, indicating how frequently each comment was true for you during the past seven days due to imagining the future*”. Items included: “Pictures about the future popped into my mind” (intrusive pre-experiencing), “I tried not to think about the future” (avoidance), and “I had waves of strong feelings about the future” (hyperarousal). Each item was anchored on a 5-point scale. In scoring the IFES, the primary variable is IFES total score which is the summation of the responses to the 24 items.

#### Barratt Impulsiveness Scale (BIS) (24)

The BIS, a 30-item self-report measure, assessed three aspects of impulsivity: attentional (rapid, unstable thoughts and lack of cognitive patience), motor (a tendency for impetuous action) and non-planning (lack of future orientation).

#### National Adult Reading Test (NART) (25)

The NART is a reading test consisting of 50 short, irregularly spelled words (e.g., debt, facade, idyll) which was used as a measure of premorbid intelligence quotient (IQ) (28).

### Analysis of data

Analyses were carried out using SPSS 15.0 for Windows (SPSS Inc., Chicago, IL, USA). Groups were compared using independent *t*-tests for continuous data and chi-square tests for categorical data. Within group comparisons were conducted using paired-samples *t*-tests. Effect sizes (Cohen's *d* for continuous data and Cramer's phi for categorical data) were calculated for all comparisons with the exception of demographic data. Participant descriptions of their appraisals of their most significant flashforward images and their responses to these flashforwards were analysed using content analysis (29) to identify key themes. Themes were elicited using two methods: empirical (new themes emerging from the data) and *a priori* (categories specified from existing research). A second rater coded 25% of the data to provide a reliability check. A high overall level of agreement was obtained between raters (Cohen's *k* =0.89, range: 0.79–1.00). Discrepancies in coding between the raters were discussed and final categories were jointly allocated.

## Results

### Demographic and clinical measures

Demographic and clinical data are shown in Tables 1 and 2, alongside statistical comparisons between groups. No differences were observed between the groups for age, premorbid IQ, the proportion of males, level of educational attainment or previous suicide attempts. The groups did not differ in current depressed mood (BDI), current mania rating (AMRS) or state anxiety (STAI-S). The bipolar group had higher suicidality ratings (BSSw screening score) than the unipolar group, had been hospitalized a greater number of times, and had greater levels of impulsivity (BIS). For both groups the number of participants with comorbid anxiety disorders and number of episodes of depression experienced are detailed in Table 2 together with the number of episodes of hypo(mania) experienced by the bipolar group.

**Table 1:** Demographic characteristics of participants in the bipolar and unipolar groups

	Bipolar (n = 20)	Unipolar (n = 20)	Statistic	p-value
Gender, n			χ^2^(1)< 0.01	1.00
Male	10	10		
Female	10	10		
Age, years, mean (SD)	38.7 (11.1)	37.9 (16.2)	*t*(38) = 0.17	0.87
Premorbid IQ^a^, mean (SD)	119.4 (6.4)	116.9 (6.0)	*t*(38) = 1.24	0.22
Ethnic origin, n			χ^2^(1)=2.11	0.35
White British	17	19		
White Irish	1	1		
White other	2	0		
Education, n			χ^2^(1)=8.78	0.19
Left school	0	3		
GCSE/ O-levels	2	1		
Vocational qualification^b^	1	2		
A-levels	1	3		
Professional qualification^c^	4	0		
Bachelor degree	7	7		
Higher degree	5	4		
Occupation, n			χ^2^(1)= 6.68	0.21
Employed full time	6	8		
Employed part time	3	3		
Unemployed	6	1		
Homemaker	1	0		
Student	4	7		
Retired	0	1		
Marital status, n			χ^2^(1) = 0.83	0.65
Single	10	10		
Married/cohabiting	5	7		
Separated/divorced	5	3		

a Assessed using the National Adult Reading Test (NART).

b Includes National Vocation Qualification, Business and Technology Education, and counselling diploma.

c Includes teaching certificate and nursing qualification.

SD = standard deviation; GCSE = General Certificate of Secondary Education.

**Table 2:** Mean (SD) scores for participants in the bipolar and unipolar groups on clinical measurements, and between-group comparisons

	Bipolar (n = 20) Mean (SD)		Unipolar (n = 20) Mean (SD)		*t*	p-value	*d*^a^
Beck Depression Inventory		27.25 (11.89)		27.30 (7.86)	0.02	0.99	< 0.01
Altman Mania Rating Scale		2.40 (2.76)		2.15 (2.23)	3.15	0.75	0.10
STAI-S^b^		45.95 (13.57)		42.35 (10.04)	0.95	0.35	0.30
Beck Suicide Scale–worst ever^c^		8.30 (1.78)		5.80 (2.65)	3.50	0.01	1.11
Barratt Impulsiveness Scale		71.70 (8.10)		65.90 (8.64)	2.19	0.04	0.69
Age at onset of disorder, years		17.35 (6.75)		22.15 (9.42)	1.77	0.08	0.59
Number of hospitalizations		2.65 (4.49)		0.30 (0.92)	2.29	0.03	0.73
Number of suicide attempts		1.15 (1.09)		0.70 (1.30)	1.18	0.24	0.38
	n		n		χ^2^(1)		
Comorbid anxiety disorder	12	0.85 (0.81)	6	0.30 (0.47)	3.63	0.11	
Number of episodes of depression					6.30	0.11	
1–5	3	4.00 (1.00)	10	2.90 (1.37)			
6–10	2	7.00 (1.41)	0	0.00 (0.00)			
11–20	1	16.00 (0.00)	2	15.50 (6.36)			
Too many to count	9	–	2	–			
Number of episodes of (hypo)mania							
1–5	4	3.25 (0.96)					
6–10	1	8.00 (0.00)					
11–20	4	18.25 (5.38)					
Too many to count	5	–					

STAI-S = Spielberger State-Trait Anxiety Inventory–State version.

a Cohen's *d* is a standardized measure of effect size in which values between 0.2 and 0.3 are taken to indicate *small* effect, values around 0.5 indicate a *medium* effect, and values ≥ 0.8 indicate a *large* effect (41).

b Rating of anxiety level in research session.

c Screening score.

## Suicidal images: flashforwards

In addition to verbal suicidal ideation, all participants in the bipolar and unipolar groups reported intrusive suicide-related images at the time when feeling at their most despairing or suicidal (see below for examples; data for all the bipolar disorder participants can be found in Table 3).

**Table 3:** Most significant flashforward image reported by bipolar group when at their most suicidal, alongside the appraisal of image and response to image

Participant no. and diagnosis	Content of image	Appraisal of image	Response to image
1. BP-I	Visualized harming myself with a razor	Makes me feel I'm going to die	Want to curl up in a ball and hibernate
2. BP-II	I imagined hanging myself from a tree on the path I regularly take to the shops	Hope that it will end mine and my family's pain	Made me want to get on with it [suicide]
3. BP-I	Image of rolling up sleeve and taking a knife or broken glass to cut wrists vertically, as there is greater chance of success	Things would be over, it would be something I could do to get release	When images were frequent would get tattoos: the pain of needles was a distraction
4. BP-I	Thinking of picking up a gun and blowing my brains out	Total escape. The easiest way to die. A way of being in control when you're out of control	Made me want to get a gun
5. BP-I	Taking an overdose	An end to all the rubbish. Going from turmoil to peace	Take tablets to find calm
6. Cyclothymia	My family identifying my body, which has gun-shot wounds. My face is ashen-coloured	Images of family were reasons not to do it, therefore, they were unwelcome. Meant there was something to stick around for	Made me not want to do it, not use the gun, not pull the trigger
7. BP-II	Sticking a gun to my head and finding the right place of my brain to hit so the first shot would kill me	I'm going to get out of this—a solution	Want to do it
8. BP-I	Imagining pills all together, how to get more pills	An escape	Wanting it to happen
9. BP-II	Mental image of where on my thigh I would have to stab myself to maximize my chances of hitting my femoral artery and bleeding to death	An escape route, a way out	Wanted to do it
10. BP-II	Image of her children crying	Suicide is not an option	Wanted to do it but could not because of effect on family
11. BP-II	Imagining self finally going to sleep	Control	I wanted to get on with it, to finish, to end and leave once and for all
12. BP-II	Me with an open bottle of white tablets of different shapes and sizes	It was a way of working it through—could I put these tablets in my mouth? Could I ingest them?	Made me think suicide was not a good idea. I had a conviction I would make a mess of it
13. BP-I	Slitting wrist, holding a blade by my vein and running it down and watching the blood pour out	An escape from everything	Avoid everybody, hide away and cry
14. BP-I	Being in trouble with criminals. Being beaten up, tortured. This is an image I have experienced when suicidal and suffering from psychotic depression	Things will get worse, something more unbearable will happen. I won't be able to stand it	Wanted to end it all so I could get away from what I felt was my lot in life
15. BP-II	I imagine picking up a knife in a kitchen, then lying slouched up against a fridge with blood everywhere	This is what I deserve. I will prove to myself that I can do this	The image motivated me to move it into reality
16. BP-I	Deliberately careening off the road and crashing car	If I do this there will be no going back	Images snapped me out of the suicidal thoughts, made me want to grip the steering wheel tighter
17. BP-I	Walking through a meadow at dusk, seeing myself getting into the water and then drowning	Something must be badly wrong. Concerned that might act impulsively on the image	Made me want to go into the hospital and seek some sort of shelter to keep the images at bay
18. BP-I	Vivid image of a patient who I was in the hospital while cutting their wrists in the bathroom. The image had a religious quality to it	Other people had the strength of mind to commit suicide—it's not just me that feels this way	Image made me want to kill myself
19. BP-I	Image of hitting head against a glass window, trying to break skull	Wanted to try and destroy the feelings of self-hatred	Image made me want to commit suicide
20. BP-I	Standing on a wall of a bridge. Imagining jumping off and drowning	I will be released from all of this, all of these thoughts	Wanted to do it, but at same time wanted to fight the urge to act

BP-I = bipolar I disorder; BP-II = bipolar II disorder.

- I had a mental image of where on my thigh I would have to stab myself to maximize my chances of hitting my femoral artery and bleeding to death (*participant with bipolar II disorder*).
- Walking through a meadow to the river at dusk, seeing myself getting into the water and drowning (*participant with bipolar I disorder*).
- Image of sticking a gun to my head or in my mouth. Finding the right part of my brain to hit so the first shot would kill me (*participant with bipolar II disorder*).
- Image of a rope in my hands followed by an observer perspective image of myself hanging from a tree, swaying in the breeze (*participant with major depressive disorder*).
- Sitting up on a cliff in my car, then my car being in the water with a breakdown lorry pulling it out. The sea was glistening and welcoming (*participant with major depressive disorder in partial remission*).
- Image of hurling myself off my balcony … maybe ten or twenty feet below was a tiled floor (*participant with dysthymic disorder*).

### Comparison of suicidal cognitions between bipolar and unipolar groups

Suicidal cognition data and the relevant between-group statistical comparisons are presented in Table 4.

**Table 4:** Mean (SD) of ratings from the suicidal cognition interview and general imagery scales for the bipolar and unipolar groups, and statistics for between-groups comparisons^a^

Variable	Bipolar (n = 20)	Unipolar (n = 20)	*t*	p-value^b^	*d*^c^
Suicidal images^d^					
Preoccupation	8.00 (1.49)	6.90 (1.83)	2.09	0.04	0.66
Compellingness	7.50 (2.40)	5.55 (2.86)	2.34	0.03	0.74
Associated distress	5.05 (3.71)	4.45 (3.15)	0.55	0.59	0.09
Associated comfort	5.65 (3.41)	4.90 (2.95)	0.74	0.74	0.24
Suicidal verbal thoughts^d^					
Preoccupation	5.30 (2.83)	4.55 (2.28)	0.92	0.36	0.29
Compellingness	5.80 (3.14)	4.95 (2.50)	0.95	0.35	0.30
Spontaneous Use of Imagery Scale	47.00 (7.54)	41.50 (7.13)	2.37	0.02	0.75
Impact of Future Events Scale	49.35 (13.74)	33.90 (12.33)	3.74	0.001	1.18

a For statistical comparisons within groups, see main text.

b To check that differences between results were not due to the number of hospitalizations (an indication of severity of psychopathology), we transformed this variable into categorical data (hospitalized/not hospitalized). We then performed ANCOVAs for each of the above comparisons using the categorical hospitalization data as a covariate. All significant differences between groups remained significant (all p < 0.03) with the exception of the Spontaneous Use of Imagery Scale score which became a trend level difference (p = 0.07). There were no changes in the direction of nonsignificant comparisons (all p > 0.15).

c Cohen's *d* is a standardized measure of effect size in which values between 0.2 and 0.3 are taken to indicate *small* effect, values around 0.5 indicate a *medium* effect, and values ≥ 0.8 indicate a *large* effect (41).

d Ratings were made on 9-point scales (1 = not at all to 9 = all the time/completely compelling/extremely distressing/extremely comforting).

#### Flashforwards imagery

Compared to the unipolar group the bipolar group reported significantly greater preoccupation with suicide-related flashforward images at the times when they felt at their most despairing or suicidal. The bipolar group also rated their flashforward images as significantly more compelling than the unipolar group.

#### Verbal thoughts

No between-group differences were found for preoccupation with verbal thoughts related to suicide or for the compellingness of these verbal thoughts.

### Comparison of flashforward images versus verbal cognitions within each clinical group separately

Suicidal cognition data are presented in Table 4 (and the within-groups comparisons in the text below rather than in the table). Ratings of flashforward images and verbal thoughts related to suicide were compared within groups. For the times when participants felt at their most despairing or suicidal, both clinical groups reported a greater preoccupation with flashforward images than suicide-related verbal thoughts (bipolar group: paired *t*-test = 3.55, df = 19, p *=* 0.002, *d* =1.19; unipolar group: paired *t*-test = 4.31, df = 19, p *<* 0.001, *d* =1.14). There was a statistical trend for the bipolar group to report the flashforward images as more compelling than suicide-related verbal thoughts (paired *t-*test = 1.81, df = 19, p = 0.09, *d* =0.47). No difference in ratings of compellingness between the two types of cognitions was found for the unipolar group (*t* <1.0).

### Detailed investigation of flashforwards

#### Participant responses to experiencing flashforwards

Responses to flashforward imagery for the bipolar disorder participants are described in Table 3. Thus, in response to the question “What did the [flashforward] image make you want to do?”, five themes emerged: take action to complete suicide, not take action to complete suicide, behavioural distraction, cognitive distraction/ suppression, and social/ behavioural avoidance. Significantly more participants in the bipolar group (13/20 participants) reported that the flashforward made them want to take action to complete suicide than the unipolar group (5/20), χ^2^=6.47, df = 1, p = 0.03, ϕ= 0.40^1^. For example, a participant in the bipolar group described an image of “picking up a gun and blowing my brains out”. The participant's response to the image was to “want to get a gun”. The participant had spent significant amounts of time on the internet researching how to acquire a gun (see Table 3). There was a trend for the unipolar group to use more cognitive distraction/ suppression strategies in response to the images (3/20 participants) than the bipolar group (0/20 participants), χ^2^=3.24, df = 1, p = 0.07, ϕ= 0.29. Other themes did not differ in frequency between groups (for all comparisons p > 0.20).

#### Participant appraisals of flashforwards

Appraisals of flashforward imagery for the bipolar group are given in Table 3. Participant appraisals of flashforwards were highly idiosyncratic but could be grouped under one of four broad themes: metacognitive interpretations (e.g., “I realised that things must be bad for me to be thinking that way”), suicide as a solution or coping strategy (e.g., “taking tablets would be a way of finding calmness”), ambivalence about suicide (e.g., “it put things into perspective … it made me realise that the way I looked at things was very warped”), and self-cognitions (e.g., “I am selfish”). There were no differences in the prevalence of these themes between groups (for all comparisons p > 0.15).

#### Distress and comfort associated with flashforwards

See Table 4 for ratings of distress and comfort, and corresponding statistical comparisons between bipolar and unipolar groups. There were no differences between the groups on ratings of distress or comfort associated with the flashforward images (for both comparisons p ≥ 0.59). In addition, no differences in distress or comfort associated with the flashforwards were found within groups, suggesting that, for both groups, the suicidal imagery was as comforting as it was distressing (bipolar group: paired *t*-test = 0.42, df = 19, p *=* 0.68; unipolar group: paired *t*-test = 0.39, df = 19, p *=* 0.70). For example, a participant in the bipolar group described an image of “rolling up my sleeve and taking a knife or broken glass to cut my wrists vertically, as there is a greater chance of success”. This image was associated with both relief and fear.

#### General imagery use in bipolar group versus unipolar group

Scores and statistical comparisons for the trait imagery measures of general imagery use are displayed in Table 4. Compared to the unipolar group, the bipolar group scored higher on both the SUIS (22) and the IFES (27), indicating greater trait and prospective imagery use in the bipolar group than the unipolar group for imagery other than that related to suicide.

## Discussion

We have compared imagery and verbal thoughts related to suicide in bipolar disorder and unipolar depression: both groups were selected for past suicidality. Replicating previous work, the unipolar group all reported experiencing imagery related to suicide, that is, flashforwards (3). As we had predicted, and for the first time, we found that all the participants with bipolar disorder also reported such imagery. Further, compared with the unipolar group, the bipolar group reported greater preoccupation with suicide-related images. The bipolar group rated their flashforward images as being significantly more compelling than did the unipolar group. These findings were unlikely simply to be due to a response bias in the bipolar group as there were no such between-group differences for the equivalent verbal thoughts related to suicide. Thus, strikingly, the bipolar group was more than twice as likely to report in the interview that the images made them want to take action to complete suicide.

As predicted, the bipolar group indeed had greater trait propensity to use mental imagery in general (i.e., non-suicidal imagery), in line with our recent cognitive model of the disorder (13); this underscores their greater preoccupation with images specifically relating to suicide. The psychological literature has shown that imagining a behaviour is causally linked to then performing that behaviour (6, 31). Our bipolar disorder sample, as predicted, specifically indicated an increased desire to act on their flashforwards and had higher scores on a measure of impulsivity. On theory-driven grounds this finding could be linked to risk for completed suicide, and is a possible contributing mechanism to the high rates of suicide found in bipolar disorder (1). However, participants in both suicidal groups reported a preponderance of flashforwards to suicide relative to verbal thoughts of suicide, so further exploration of imagery of future events might provide a novel and much-needed general target for intervention.

In both groups, the suicidal imagery was as comforting as it was distressing. Interestingly, there are also many vivid artistic representations of the act of suicide which date back at least to the 15^th^ century (32), in which suicide can be depicted in a positive light. For example, the painting *Ophelia* by John Everett Millais (1829–1896) is a romantic depiction of a young woman who is drowning herself (33). A comparison of flashforward images with their verbal counterparts showed that both groups reported greater preoccupation with these suicide-related images rather than words (verbal thoughts or narratives about suicide).

It has been suggested that engaging in suicidal fantasies may serve as an emotion regulation strategy (e.g., 4, 34), and so that the generation of suicidal flashforwards is an *active* process. In line with this argument, it seems that flashforward images can function as a means to escape or seek comfort from the examples reported by the bipolar disorder participants in our sample. However, flashforwards may also be the result of a more *passive*, automatic process in which the image is unwanted and comes to mind unbidden. The type of process associated with the flashforward may also be linked to whether the image is experienced as comforting or distressing.

The degree of comfort and distress associated with the suicidal flashforwards may vary according to whether the content of the imagery is consistent with the goals of the individual or represents a conflict. For example, if an individual is distressed by the prospect of living and comforted by the idea of killing themselves, ensuing comforting emotions could be considered to be consistent with the content of suicidal flashforward imagery, potentially increasing suicide risk. However, if a patient reports both fear and comfort associated with the idea of killing oneself then the flashforward imagery may be experienced as conflictual, and represent ambivalence about suicide. Future studies could use a functional/conflicted goals analysis to investigate these aspects of suicidal imagery further as it may be a useful tool in assessing clinical levels of risk.

Our hypothesis remains that the use of imagery is particularly associated with bipolarity as a dimension of experience and psychopathology, and this is supported by this study. We would expect a contrast with other clinical groups such as schizophrenia with reduced rather than increased levels of imagery for the future compared to controls (35). It will be of great interest to investigate whether flashforwards to suicide are present in other disorders characterized by mood instability and suicidal thinking, such as borderline personality disorder, schizoaffective disorder and so forth.

### Limitations

Comparisons between bipolar and unipolar groups pose problems of experimental design because it is not entirely clear which clinical measures should be matched/controlled and which allowed to be different (because the bipolar diagnosis implies they should be different). The characteristics of much larger clinical samples suggest, for example, that bipolar depression tends to be associated with more frequent episodes and admissions and higher impulsivity as observed here. A recent consensus review of bipolar depression (36) emphasized the gradient between bipolar and unipolar depression and the relative absence of a clear boundary between the two. Moreover, it may be generally true that a bipolar diagnosis is associated on average with more severe psychopathology. The present study cannot decide whether higher suicidal imagery should be attributed simply to severity rather than diagnosis *per se*, and clinically this may not matter.

The groups did not differ in current depressed mood, current mania, state anxiety, or IQ, all of which might have influenced the results. There was a higher lifetime illness intensity in the bipolar group, with more hospitalizations, more episodes of illness, higher impulsivity scores and a tendency towards greater anxiety [see also (37)]. Such features are present in larger representative samples of bipolar disorder patients. The homogeneity and size of our sample prevented investigation of relationships between, for example, appraisal of imagery and severity of client psychopathology. This would be interesting to explore in the future using logistic regression methods in a larger sample. Finally, we excluded severely suicidal participants in part to satisfy the conventions of our local ethics committee. Future research, perhaps in an inpatient setting (cf., 38), could examine an actively suicidal population in more detail.

In a cross-sectional design the causal direction of any association will remain uncertain, but investigating a possible relationship between number of hospitalizations and flashforward imagery may be of particular interest. Thus, if such imagery is causally related to future behaviour, high levels of suicidal imagery would predict an increased risk of hospitalization. Larger prospective studies of at-risk individuals will be needed to partial out this or other causal effects in the evolution of mood disorder.

Lastly, our finding of very specific images associated in memory with suicidal acts appears incompatible with studies showing that bipolar disorder patients have overgeneral memories compared with controls (39, 40). However, such overgenerality in the narrative domain may be attributable to processes for keeping specific distressing images at bay (37).

Interestingly, the vast majority of participants spontaneously remarked that they had never discussed their suicidal images with their clinicians. This may be because typically clinicians ask for verbal narrative when eliciting suicidal ideation (“Do you have any thoughts of killing yourself?”), rather than specifically enquiring about suicidal imagery. However, we did not assess this formally, and a replication study should aim to investigate this.

## Conclusions

Mental imagery of suicide may be an important but neglected feature of suicidal ideation, particularly in bipolar disorder. Suicidal risk assessment is an area that requires fresh ideas to improve clinical care. Because suicide rates are elevated in bipolar disorder, enhanced features of suicidal cognition in this group are of particular interest. If our observations and their implications are confirmed, exploration of suicidal imagery will warrant inclusion in clinical assessment procedures. Rather than simply asking “Do you have thoughts about killing yourself?; do you have a plan?”, we predict that it may be useful to add questions about imagery such as “Do you imagine killing yourself?; have you rehearsed how to do this in your mind's eye?”. Confirmation of such experiences promises clinical utility for an area in need of innovation.

## Footnotes

1 Cramer's ϕ is a standardized measure of effect size in which a value of 0.10 is taken to indicate *small* effect, a value of 0.30 indicates a *medium* effect, and 0.50 indicates a *large* effect (30).
